# Peroxisome Proliferator Activator Receptor (PPAR)-**γ** Ligand, but Not PPAR-**α**, Ameliorates Cyclophosphamide-Induced Oxidative Stress and Inflammation in Rat Liver

**DOI:** 10.1155/2014/626319

**Published:** 2014-04-02

**Authors:** Azza A. K. El-Sheikh, Rehab A. Rifaai

**Affiliations:** ^1^Department of Pharmacology, Faculty of Medicine, Minia University, Minia 61511, Egypt; ^2^Department of Histology, Faculty of Medicine, Minia University, Minia 61511, Egypt

## Abstract

Hepatoprotective potential of peroxisome proliferator activator receptor (PPAR)-**α** and -**γ** agonists, fenofibrate (FEN), and pioglitazone (PIO), respectively, against cyclophosphamide (CP)-induced toxicity has been investigated in rat. FEN and PIO (150 and 10 mg/kg/day, resp.) were given orally for 4 weeks. In separate groups, CP (150 mg/kg, i.p.) was injected as a single dose 5 days before the end of experiment, with or without either PPAR agonist. CP induced hepatotoxicity, as it caused histopathological alterations, with increased serum alanine and aspartate transaminases, total bilirubin, albumin, alkaline phosphatase and lactate dehydrogenase. CP caused hepatic oxidative stress, indicated by decrease in tissue reduced glutathione, with increase in malondialdehyde and nitric oxide levels. CP also caused decrease in hepatic antioxidant enzyme levels, including catalase, superoxide dismutase, glutathione peroxidase, and glutathione S-transferase. Furthermore, CP increased serum and hepatic levels of the inflammatory marker tumor necrosis factor (TNF)-**α**, evaluated using ELISA. Preadministration of PIO, but not FEN, prior to CP challenge improved hepatic function and histology, and significantly reversed oxidative and inflammatory parameters. In conclusion, activation of PPAR-**γ**, but not PPAR-**α**, conferred protection against CP-induced hepatotoxicity, via activation of antioxidant and anti-inflammatory mechanisms, and may serve as supplement during CP chemotherapy.

## 1. Introduction

Cyclophosphamide (CP) is a synthetic alkylating agent that has for long been successfully used in treatment of cancer and autoimmune diseases, as well as in the prevention of organ transplantation rejection [1]. Despite of its tumor selectivity and wide range of clinical applications, CP is known to cause multiorgan damage that result in severe morbidity and might end fatally [2]. Most reports focused on studying CP-induced cardio- and gonadotoxicity [3–5], with much lesser attention to hepatotoxicity [6]. CP-induced hepatotoxicity may occur at high chemotherapeutic dosage [7] or even at lower concentrations attained during treating patients with autoimmune diseases [8, 9]. To date, the mechanisms involved in CP-induced hepatotoxicity are not completely clarified. It has been proposed that administration of CP might cause impairment of cellular respiration due to damage of mitochondrial energy converting mechanisms [10], which may interfere with hepatic intracellular oxidant/antioxidant balance and lead to accumulation of reactive oxygen species [11]. The resultant oxidative stress may then trigger nuclear factor-*κ*B (NF-*κ*B) inflammatory pathway, which increases hepatic intracellular proinflammatory cytokines as tumor necrosis factor (TNF)-*α* [12].

Fenofibrate (FEN) and pioglitazone (PIO) are peroxisome proliferator activator receptor (PPAR)-*α* and -*γ* agonists that are used as antihyperlipidemic [13] and antidiabetic agents [14], respectively. We have recently shown that FEN and PIO possessed comparable antioxidant, but not anti-inflammatory, properties, and that they confer nephroprotection against toxicity of another anticancer drug, namely, methotrexate [15]. Still, the hepatic safety of these PPAR ligands has been controversial. FEN was reported to have hepatic favorable effects in some studies [16], whereas in others, FEN was reported to cause fatty liver in mice [17] and acute cholestatic hepatitis in humans [18]. Hepatic safety of PIO is also still controversial. While long term follow-up in a 3-year human study declared that PIO have no substantial hazard on the liver [19]; another study reported that PIO might be the cause of sporadic cases of liver failure [20]. Interestingly, both FEN [21, 22] and PIO [23, 24] were suggested to modulate hepatic oxidant/antioxidant parameters and inflammatory cytokines, which may suggest that they confer hepatoprotective effects. The objective of this study is to establish the potential use of PPAR-*α* and -*γ* agonists, FEN, and PIO, respectively, as supplementary adjuvant to protect against CP-induced hepatotoxicity and to investigate the pharmacological mechanisms involved.

## 2. Materials and Methods

### 2.1. Chemicals

FEN and PIO were kind gifts from Sigma Pharmaceutical Industries and Medical Union Pharmaceuticals (Egypt), respectively. CP was purchased from Baxter Oncology (Germany). Kits for examining total bilirubin, albumin, alanine transaminase (ALT), aspartate transaminase (AST), alkaline phosphatase (ALP), and lactate dehydrogenase (LDH) in serum, as well as reduced glutathione (GSH), superoxide dismutase (SOD), catalase (CAT), glutathione peroxidase (GPX), and glutathione* S*-transferase (GST) in liver homogenate were purchased from Biodiagnostic (Egypt). TNF-*α* enzyme-linked immunosorbent assay (ELISA) kit was purchased from WKEA-Med supplies Corp. (China).

### 2.2. Experimental Design

Forty-eight adult male albino rats (180–220 g) were purchased from the National Research Centre (Giza, Egypt). Rats were placed in the standard animal facility throughout the experiments, housed 4 animals per cage. Tap water and laboratory chow were freely accessed. The study protocol was consistent with the guidelines and approved by the Research Ethical Committee of Faculty of Medicine, Minia University. For 2 weeks before the start of experiments, animals were left to acclimatize. After acclimatization period, animals were divided into 6 groups (*n* = 8 each): control untreated group, FEN- and PIO-treated groups receiving single daily oral dose of 150 and 10 mg/kg/day of FEN and PIO, respectively [24, 25], by gastric gavage for 4 weeks, and CP-treated group receiving a single i.p. dose of 150 mg/kg 5 days before the end of the experiment [26]. Two other groups of combined CP/FEN and CP/PIO received CP, FEN, and PIO treatments as previously indicated. Total rat body weights were recorded before the start and at the end of the 4-week experiment. Percent of change in body weight was evaluated by calculating the percent of the difference between final and initial weights of each animal group compared to control.

### 2.3. Sample Preparation and Histopathological Examination of Liver

At the end of the 4-week experiment, rats were sacrificed. Venous blood samples were collected from the jugular vein and centrifuged at 5000 rpm for 15 min and serum was collected and stored at −80°C till used. Liver was rapidly excised and weighed. Liver sections were taken for histopathological examination and the rest of the liver tissue was snap-frozen in liquid nitrogen and kept at −80°C. For histopathology, liver specimens were fixed in 10% buffered neutral formalin solution, dehydrated in gradual ethanol (70–100%), cleared in xylene, and embedded in paraffin. Five *μ*m thick paraffin sections were prepared and then stained with hematoxylin and eosin (H&E) dyes [27]. Stained slides were microscopically analyzed using light microscopy (Olympus CX41). For scoring different histopathological parameters, 5 sections from each rat liver were examined for necrotic degeneration, fatty changes, and inflammatory cellular infiltration. Histopathological damage was graded according to a semiquantitative scoring as no change, mild, moderate, or severe [28]. To prepare tissue homogenate, livers were homogenized (Glas-Col homogenizer) and a 20% w/v homogenate was prepared in ice-cold phosphate buffer (0.01 M, pH 7.4). The homogenate was centrifuged at 3000 rpm for 20 min and the supernatant was then divided over several containers to avoid sample thawing and refreezing and was kept at −80°C till used.

### 2.4. Evaluation of Serum Markers of Liver Function and Oxidant/Antioxidant Markers in Liver Homogenate

Using commercially available colorimetric diagnostic kits, assessment of liver function and hepatotoxicity were done by determination of total bilirubin, albumin, ALT, AST, ALP, and LDH in serum, according to the manufacturer's instructions. Biochemical oxidative stress markers were determined in liver homogenate, including GSH, nitric oxide (NO), and lipid peroxide content assessed by malondialdehyde (MDA) level. A spectrophotometric kit was used for assessment of GSH. In Brief, the method is based on that the sulfhydryl component of GSH reacts with 5,5-dithio-bis-2-nitrobenzoic acid (Ellman's reagent) producing 5-thio-2-nitrobenzoic acid having a yellow color that was measured colorimetrically at 405 nm (Beckman DU-64 UV/VIS spectrophotometer). Results were expressed as *μ*mol/g tissue. For NO, the stable oxidation end products of NO, nitrite and nitrate, were used as an index of NO production, as NO has an extremely short half-life of few seconds, as it is readily oxidized to nitrite then to nitrate. The method used was based on Griess reaction that depends on measurement of total nitrites at 540 nm after the conversion of nitrate to nitrite by copperized cadmium granules [29], using nitric acid as a standard. Results were expressed as nmol/0.1 g tissue. Tissue content of lipid peroxides was assessed via biochemical evaluation of thiobarbituric acid reacting substance through spectrophotometric measurement of color at 535 nm, using 1,1,3,3-tetramethoxypropane as standard. The results were expressed as equivalents of malondialdehyde (MDA) in tissue homogenate in nmol/g tissue [30].

### 2.5. Determination of Hepatic Antioxidant Enzymatic Activity

Antioxidant enzymatic activity of CAT, SOD, GPX, and GST were determined in hepatic tissue homogenate using commercial kits according to the manufacturer instructions. Briefly, hepatic CAT activity was calculated from the rate of decomposition of H_2_O_2_ at 510 nm after the addition of liver homogenate and results were expressed as U/g tissue. The SOD determination assay depended on the ability of SOD enzyme to inhibit the phenazine methosulphate-mediated reduction of nitroblue tetrazolium dye. The change in absorbance at 560 nm was measured over 5 min. SOD activity results were expressed in U/0.1 g tissue. Hepatic GPX and GST activities were evaluated spectrophotometrically using reduced glutathione as substrate by addition of liver homogenate measured at 340 nm. GPX and GST results were expressed in U/g tissue and U/mg tissue, respectively.

### 2.6. Assessment of Proinflammatory Cytokine, TNF-*α*, in Serum and Liver Homogenate

According to manufacturer's instructions, 10 *μ*L of serum or liver homogenate were dispensed in 40 *μ*L of sample diluent solution, mixed, and incubated for 30 min at 37°C. After the first incubation, the plate was washed five times with 30-fold diluted wash buffer and then dried. 50 *μ*L enzyme conjugate was added to each well, incubated, then washed as previously described. After drying the plate, 50 *μ*L of substrate A and 50 *μ*L of substrate B were added to each well and the plate was incubated for 15 min at 37°C. The reaction was stopped by adding 50 *μ*L stop solution. The plate was then read using ELISA plate reader at 450 nm.

### 2.7. Statistical Analysis

The data was analyzed by one way ANOVA followed by Dunnett Multiple Comparison Test. The values are represented as means ± S.E.M. All statistical analysis was done using GraphPad Prism (GraphPad Prism software, 2011). The differences were considered significant when the calculated *P* value is less than 0.05.

## 3. Results

### 3.1. Effect of FEN and PIO on Hepatic Histopathological Findings in CP-Treated Rat

**Figure 1:**
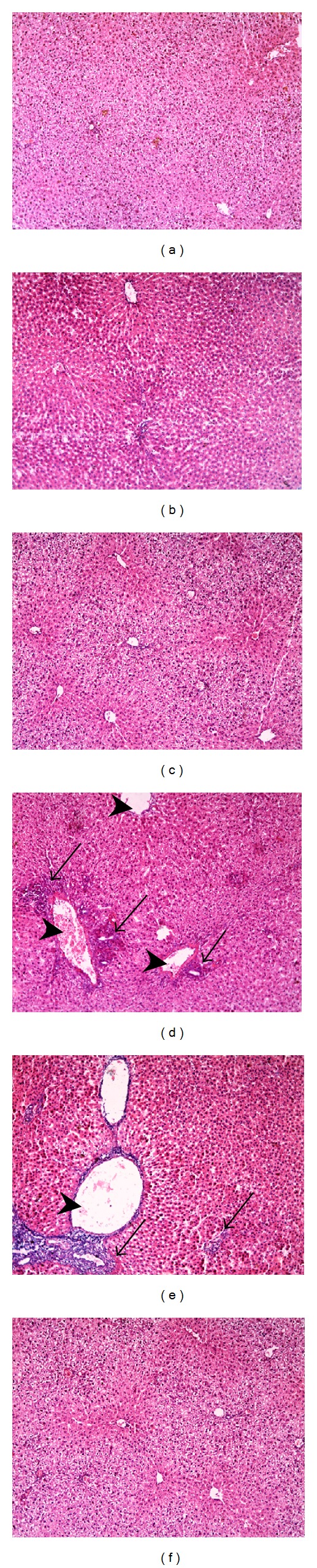
Effect of fenofibrate (FEN) and pioglitazone (PIO) on liver histopathological profile in cyclophosphamide (CP)-treated rats. Representative photomicrographs of liver from: ((a), (b), and (c)) control and FEN and PIO groups, respectively, showing no pathological changes in hepatocytes, (d) CP-treated group presenting with loss of normal hepatic architecture, congested dilated central vein, inflammatory cellular infiltration, and perivenular hepatocytic necrosis, (e) CP/FEN group showing congested dilated central veins with focal inflammatory cellular infiltration and degenerative necrotic cells, and (f) CP/PIO group demonstrating normal liver histology. Arrowhead: dilated central vein, black arrow: inflammatory cellular infiltration (×100). The histological changes were scored, and results are expressed in Table 1.

Liver sections from control, FEN, and PIO groups (Figures 1(a), 1(b), and 1(c), resp.) showed normal hepatic structure. Single administration of CP was followed by loss of normal hepatic architecture (Figure 1(d)). The central vein was dilated and congested. Sections demonstrated migration of inflammatory cells from the central vein to infiltrate the perivenular area that showed necrotic hepatocytes. FEN/CP group did not show improvement compared to CP alone, with multiple foci of degenerative necrotic cells, fatty changes, and inflammatory cellular infiltration. On the other hand, pretreatment with PIO prior to CP challenge (Figure 1(f)) caused marked improvement in liver histological picture. Scoring of histological hepatic changes is summarized in Table 1.

### 3.2. Effect of FEN and PIO on Weight Changes and Liver Functional Parameters in CP-Treated Rat

In CP-treated group, percent of change of body weight was significantly decreased, while liver/total body weight ratio was significantly increased compared to control (Table 2). Pretreatment with FEN before administration of CP did not improve either parameters, whereas pretreatment with PIO improved both parameters to level not statistically significant from control. Neither FEN nor PIO alone affected either parameter. After CP challenge, blood biochemical parameters indicative of liver function deteriorated, as evident by significant increase in serum levels of total bilirubin, ALT, AST, ALP, and LDH, with significant decrease in serum albumin (Table 2). CP/FEN group did not show any improvement in these liver functional parameters, whereas CP/PIO group demonstrated significant improvement compared to group treated with CP alone.

### 3.3. Effect of FEN and PIO on Oxidation Markers and Antioxidant Enzymes in CP-Treated Rat Liver

**Figure 2:**
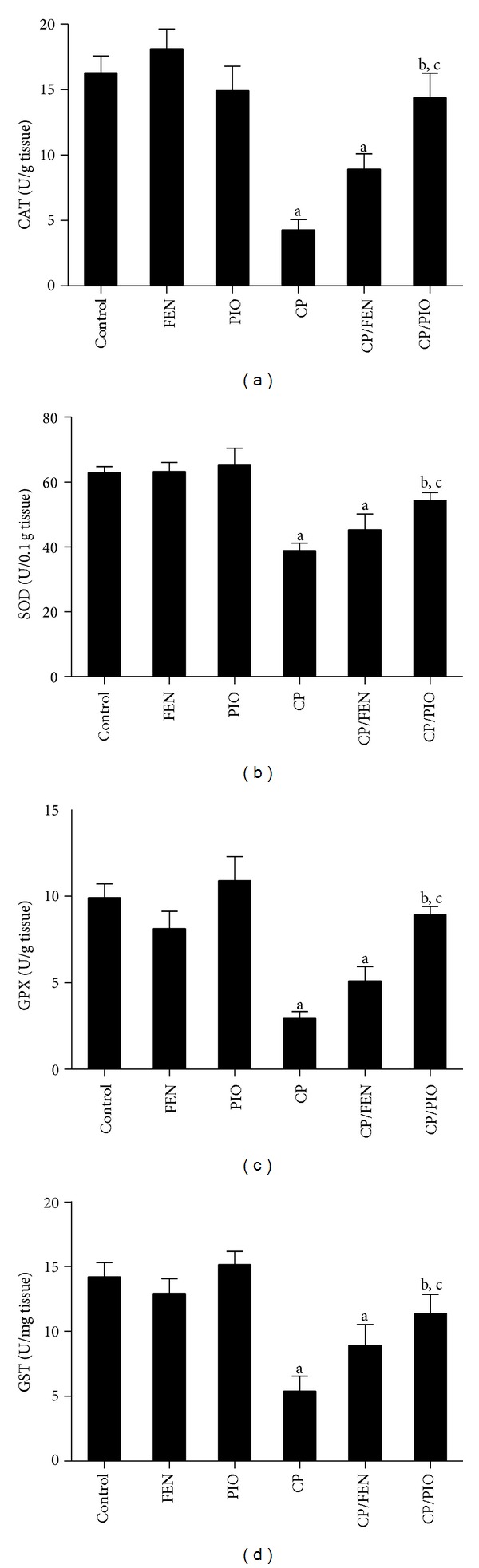
Effect of fenofibrate (FEN) and pioglitazone (PIO) on hepatic antioxidant enzyme levels in cyclophosphamide (CP)-treated rats. CAT: catalase, SOD: superoxide dismutase, GPX: glutathione peroxidase, GST: glutathione* S*-transferase. Values are representation of 8 observations as means ± SEM. Results are considered significantly different when *P*< 0.05. ^a^Significant difference compared to control, ^b^significant difference compared to CP group, ^c^no significant difference compared to control.

Table 3 depicts the effect of FEN and PIO, with or without CP challenge, on levels of hepatic GSH, NO, and MDA. CP treatment significantly decreased hepatic GSH compared to control group. FEN administration before CP failed to restore hepatic GSH level, while PIO pretreatment significantly increased GSH level compared to CP sole treatment. In addition, liver homogenate of CP group exhibited higher levels of lipid peroxidation as indicated by significantly higher levels of MDA compared to control. Liver homogenate of CP-treated group also showed significantly higher levels of NO compared to control. Pretreatment with PIO, but not FEN, succeeded in reversing MDA and NO to levels statistically significant from CP-treated group. As shown in Figure 2, the activity of hepatic antioxidant enzymes CAT, SOD, GPX, and GST were significantly less in CP group compared to control. Only pretreatment with PIO, but not FEN, significantly increase these enzymatic activities, compared to CP alone.

### 3.4. Effect of FEN and PIO on Serum and Hepatic TNF-*α* Level in CP-Treated Rat

**Figure 3:**
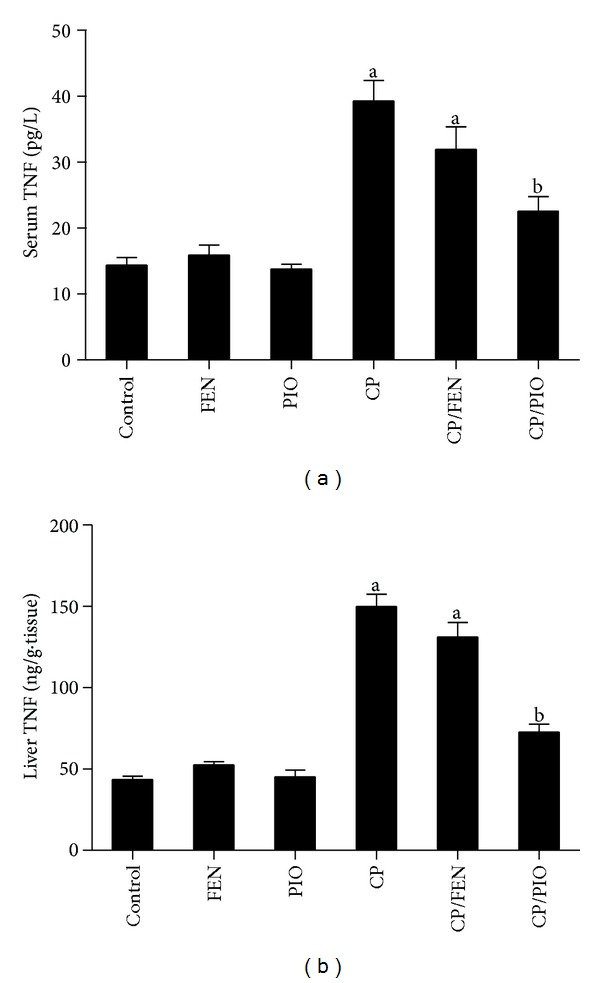
Effect of fenofibrate (FEN) and pioglitazone (PIO) on tumor necrosis factor (TNF)-*α* levels in serum and liver of cyclophosphamide (CP)-treated rats. Values are representation of 8 observations as means ± SEM. Results are considered significantly different when *P*< 0.05. ^a^Significant difference compared to control, ^b^significant difference compared to CP group.

Serum and hepatic TNF-*α* levels were significantly higher in CP-treated group than in control group (Figure 3). FEN pretreatment before CP injection did not show any statistical difference from CP alone, either for serum or hepatic TNF-*α* levels. PIO pretreatment, on the other hand, significantly decreased TNF-*α* values in serum and liver, compared to CP sole therapy.

## 4. Discussion

Hepatotoxicity due to the use of CP has been a limitation facing its use as a successful anticancer chemotherapeutic drug that possess other medical applications as treating autoimmune diseases and graft-versus-host rejection. The present* in vivo* study was designed to investigate the potential protective role of PPAR agonists against CP-induced hepatotoxicity. Our results show that the PPAR-*γ* agonist, PIO, but not the PPAR-*α* agonist, FEN, has hepatoprotective effects, through ameliorating CP-induced hepatic oxidative stress and inflammation. In the present study, CP administration caused hepatotoxicity evident by distortion of histological features and significant alteration in biochemical blood parameters indicative of liver function, which was in line with previous studies [26, 31–33].

Despite that ALT and AST are not liver-specific and their level may not by reliable reflection of the severity of hepatic damage [34], these transaminases are located cytoplasmically and are the first to be released after liver damage [35]. Similarly, increased ALP is not restrictedly a marker of liver disease, still is a very useful serum biochemical indicator of liver damage, especially cholestatic disease [36]. Likewise, LDH is another enzyme released in liver injury with low hepatic specificity, yet LDH is considered a predictor of acute liver failure [37]. Serum albumin level, on the other hand, is considered an indicator of the degree of hepatic damage, while total bilirubin level reflects cholestatic injury in rats [38].

The exact mechanisms involved in CP-induced hepatotoxicity are not yet completely clarified. Still, several recent studies suggested that oxidant/antioxidant imbalance and release of proinflammatory cytokines are, at least in part, participating mechanisms in CP-induced hepatic damage [11, 26]. In the present study, administration of CP caused decrease in antioxidant enzymatic activity of SOD, CAT, GPX, and GST, as well as alteration of markers of oxidative stress as increase in MDA and NO, with decrease in GSH and increase in level of proinflammatory cytokine, TNF-*α*.

CP is metabolically activated via hepatic microsomes forming two active metabolites, namely, phosphoramide mustard and acrolein [39]. The latter is responsible for the toxic effects of CP, as it induces free radical formation [40]. One type of these free radicals is the short-lived and highly reactive NO free radical [41]. GSH, the most powerful nonenzymatic antioxidant in the human body, is initially increased within the first 24 hours after CP assault as a part of the natural body defense mechanism against overproduction of free radical [42]. This is followed latter by depletion of GSH body stores [11, 26, 31], which fails to balance cellular oxidative status. Activity of hepatic antioxidant enzymes as SOD, CAT, GPX, and GST is also decreased during trying to overcome reactive oxygen species overproduction [11, 39]. Interestingly, GST is one of the enzymes essential for the metabolism of CP [42]. This detoxification pathway depends on conjugation of CP with glutathione, providing thiol group, catalyzed by GST. Inhibition of this pathway would increase CP serum concentration, and hence, its hepatotoxicity. The depletion of GSH and decreased activity of antioxidant enzymes are associated with increase in lipid peroxidation [39], resulting in an increase in MDA formation, a highly reactive three carbon dialdehyde which is a polyunsaturated fatty acid peroxidation and arachidonic acid metabolism byproduct. This sequence of events then triggers inflammatory process by stimulating NF-*κ*B/TNF-*α* pathway, with the increase of the proinflammatory cytokine TNF-*α*. We have recently reviewed the crosstalk between oxidative stress and NF-*κ*B/TNF-*α* pathway [43], showing that their causal/effect relationship might not be that simple.

In the present study, activation of PPAR-*α* via pretreatment with FEN did not succeed in restoring normal liver histology, improving hepatic functional parameters, reverting oxidative stress, nor inflammatory process seen after CP challenge. Still, it is noteworthy that, in the current study, administration of FEN alone, without CP, did not significantly deteriorate any of the previously mentioned parameters. In reported literature, effect of FEN on the liver varies from incriminating it of liver damage to announcing it as a hepatoprotector. For example, at one hand, some studies reported that FEN may cause variable levels of liver damage in animal models [17, 44] and humans [18, 45]. On the other hand, FEN was reported to confer hepatoprotection against acetaminophen-induced liver toxicity [21], carbon tetrachloride-induced liver cirrhosis [46], and hepatic ischemia-reperfusion [47], and had no hazardous effect on liver in chronic hemodialysis patients [48]. The mild increase in ALT and AST seen in patients receiving FEN was attributed to increased production of these enzymes, due to induction of their respective gene expression, without any underlying hepatic toxicity [49]. Similarly, studies investigating the effect of FEN on antioxidant enzymes varied from declaring its antioxidant properties [50], announcing its lack of effect on these enzymes [51], or claiming FEN as a prooxidant [52, 53]. Likewise, the effect of PPAR-*α* activation by FEN on serum and hepatic levels of the proinflammatory cytokine,; TNF-*α*, varied in different reported studies [54]. It is possible that the reported variation in antioxidant/anti-inflammatory properties of FEN is due to different dosage and/or duration regimens used in these various studies.

In the current study, the PPAR-*γ* agonist, PIO, achieved protective effects against CP-induced hepatotoxicity, as evident by repairing hepatic pathological picture, recovering liver functional enzymes, and reversing oxidative stress and inflammatory process. Despite sporadic contradictory studies [20, 55], PIO has been reported to attenuate liver injury via recovering hepatic oxidant/antioxidant balance in several animal models, as it succeeded in repairing hepatic DNA damaged due to high fat diet in mice [56], abolishing hepatic oxidative stress in alloxan-induced diabetic rabbit [57], preventing lipopolysaccharide-induced liver injury [58], and recovering liver after ischemia/reperfusion in rats [23]. In humans, PIO was demonstrated to improve hepatic functional parameters in nonalcoholic fatty liver patients [59] and in nondiabetic patients suffering from metabolic syndrome [60]. PIO has also been reported to possess hepatic anti-inflammatory properties [23, 24, 58], whose mechanism is probably via activation of PPAR-*γ* that acts as a feedback mechanism by inhibiting NF-*κ*B activation [61] and, consequently, decreasing the formation of the proinflammatory cytokine, TNF-*α*. Favorable effects of PIO have also been reported to affect organs other than the liver, as the heart [62], kidney [15, 63], and testis [64]. Future studies are necessary to prove whether these favorable beneficial effects of PIO may offer protection against CP-induced toxicity in these vital organs. Studies should also be performed to exclude interaction of PIO with CP metabolism and clearance, and hence CP's therapeutic efficacy and cytotoxicity.

## 5. Conclusion

CP has prooxidant and proinflammatory properties, which are, at least in part, the causative mechanisms of CP-induced hepatotoxicity. Unlike the PPAR-*α* agonist;, FEN, the PPAR-*γ* agonist, PIO, has hepatoprotective effect against CP-induced liver damage and might provide successful adjuvant during CP chemotherapy. The mechanisms involved include the ability of PIO to promote hepatic antioxidant capacity and ameliorate inflammation, which act in synergy to restore liver function.

## Figures and Tables

**Table 1:** Effect of fenofibrate (FEN) and pioglitazone (PIO) on histological findings in cyclophosphamide (CP)-treated rats liver.

	Degeneration and necrosis	Fatty changes	Inflammatory cell infiltration
Control	−	−	−
FEN	+	−	−
PIO	−	−	−
CP	+++	++	+++
CP/FEN	+++	+++	+++
CP/PIO	+	−	−

From each animal, 5 sections were examined and scored according to the following criteria: (−) = absent, (+) = mild, (++) = moderate, and (+++) = severe changes.

**Table 2:** Effect of fenofibrate (FEN) and pioglitazone (PIO) on change (Δ) of total body weight (wt), liver/body wt ratio, and liver function parameters in cyclophosphamide (CP)-treated rats.

	Control	FEN	PIO	CP	CP/FEN	CP/PIO
ΔBody wt (%)	100 ± 9	99 ± 9	99 ± 7	79 ± 5^a^	82 ± 4^a^	94 ± 6^b,c^
Liver/wt ratio	198.3 ± 25.8	192.4 ± 31.4	181.9 ± 29.1	314.8 ± 40.2^a^	329.2 ± 21.6^a^	201.8 ± 32.4^b,c^
Albumin (g/dL)	3.2 ± 0.7	2.9 ± 0.3	3.5 ± 0.2	1.9 ± 0.4^a^	1.7 ± 0.8^a^	2.9 ± 0.7^b,c^
Total bilirubin (mg/dL)	0.18 ± 0.03	0.22 ± 0.08	0.19 ± 0.02	0.39 ± 0.09^a^	0.36 ± 0.04^a^	0.25 ± 0.06^b,c^
ALT (U/dL)	39.9 ± 3.4	45.1 ± 8.8	35.1 ± 11.8	71.8 ± 14.2^a^	68.3 ± 9.1^a^	55.3 ± 7.8^b^
AST (U/dL)	99.1 ± 14.9	110.1 ± 12.3	95.6 ± 12.5	192.8 ± 16.3^a^	181.9 ± 11.3^a^	144.3 ± 8.3^b^
ALP (U/L)	130.5 ± 16.1	140.9 ± 18.6	122.7 ± 17.8	292.3 ± 26.2^a^	295.2 ± 21.7^a^	184.7 ± 18.9^b^
LDH (U/L)	782.2 ± 36.2	820.2 ± 38.4	799.2 ± 42.3	1452.4 ± 76.8^a^	1445.1 ± 21.7^a^	1142.9 ± 88.3^b^

Liver/wt is ratio of weight of liver divided by total body wt ∗ 1000 ratio. ALT: alanine transaminase, AST: aspartate transaminase, ALP: alkaline phosphatase, and LDH: lactate dehydrogenase. Values are representation of 8 observations as means ± SEM. Results are considered significantly different when *P* < 0.05. ^a^Significant difference compared to control, ^b^significant difference compared to CP group, ^c^no significant difference compared to control.

**Table 3:** Effect of fenofibrate (FEN) and pioglitazone (PIO) on hepatic tissue reduced glutathione (GSH), malondialdehyde (MDA), and nitric oxide (NO) levels in cyclophosphamide (CP)-treated rats.

	GSH (*μ*mol/g tissue)	MDA (nmol/g tissue)	NO (nmol/0.1 g tissue)
Control	28.9 ± 3.8	2.3 ± 0.6	18.3 ± 5.4
FEN	31.1 ± 4.3	1.9 ± 0.6	16.8 ± 2.3
PIO	26.5 ± 4.3	2.4 ± 0.7	21.5 ± 2.5
CP	16.3 ± 3.4^a^	7.9 ± 1.8^a^	64.2 ± 11.1^a^
CP/FEN	18.2 ± 3.2^a^	6.8 ± 0.4^a^	55.4 ± 12.3^a^
CP/PIO	22.8 ± 2.8^b^	5.1 ± 0.8^b^	27.9 ± 7.4^b,c^

Values are representation of 8 observations as means ± SEM. Results are considered significantly different when *P* < 0.05. ^a^Significant difference compared to control, ^b^significant difference compared to CP group, ^c^no significant difference compared to control.
